# The Texas State Society of Social Hygiene

**Published:** 1914-03

**Authors:** 


### Department of Eugenics.

Conducted by Dr. Malone Duggan and Dr. Theo. Y. Hull.

### THE TEXAS STATE SOCIETY OF SOCIAL HYGIENE.

Headquarters: San Antonio, Texas.

Dr. Malone Duggan, President, San Antonio.
Dr. F. E. Daniel, 1st Vice President, Austin.
Prof. A. Caswell Ellis, 2d Vice President, Austin.
Mrs. Eli Hertzberg, 3d Vice President, San Antonio.
Dr. Theo. Y. Hull, Secretary, San Antonio.
Mr. W. L. Evans, Treasurer, San Antonio.

EXECUTIVE COMMITTEE.

Dr. Malone Duggan, Chm.
Dr. Theo. Y. Hull, Sec'y.
Dr. W. F. McCaleb.
Hon. C. H. Jenkins.
Rev. A. W. S. Garden.
Hon. J. C. Willacy.
Mr. W. L. Evans.
Dr. Frank D. Boyd.
Dr. J. R. Nichols.
Dr. J. M. McCutchan.
Dr. W. A. King.
Dr. J. W. Torbett.
Dr. F. C. Walsh.
Dr. Joe Reuss.
Dr. Frank Paschal.

I wish to take this opportunity to thank Dr. Hull for his interesting contribution to this department during my long illness. Changes are often very welcome and beneficial, and no doubt the readers of the "Red Back" have profited by this one. Resuming again our responsibility we promise another change should we grow too tiresome.

A correct knowledge of eugenics is more needed now than ever. So much salacious literature has been sent broadcast over the country, under the name of eugenics, that there is much to be done in undoing the harmful influence that it has produced. From every quarter may be heard some obtrusive voice proclaiming himself an apostle of eugenics, whose only aim is to exploit some sex theory.

Let us keep in mind the true purpose of eugenics, which is to improve the innate quality of the race and add something to the sum of human happiness. There is nothing in its practice that is in the least sensual or ludely suggestive, nor should the principles for which it stands be confounded with abnormal sexuality. It is true, that the sex function is an important factor in its operations. Indeed, it is the essential factor. For by it procreation of the species is made possible.

The moral man is born, not made. He is moral by virtue of his heredity. Likewise the immoral man. In other words, morality or sensuality are inherent qualities transmissible from generation to generation. The force of their operations may be inhibited by favorable environments but can never be eradicated except by selective breeding. When will society learn that its chief business is to breed out undesirable qualities in the man and to breed in good ones? This is plainly the duty of the present generation. But how it should best be done is not so evident and its solution is the subject of earnest investigation on the part of the rational eugenist.

